# Supplementary material for: The portrayal of panic-buying and stockpiling in English newspapers during Covid, a mixed-method content analysis
Source: PLoS One. 2025 Feb 12;20(2):e0315142. doi: 10.1371/journal.pone.0315142 (PMC11819479; doi:10.1371/journal.pone.0315142)
Supplement: S1 File — (DOCX) [file pone.0315142.s001.docx]

S3: List of articles used in analysis, in date order.

1. Jolly J, Smithers R. UK supermarkets draw up plan to 'feed the nation' as coronavirus spreads; Plans would help with panic-buying brought on by escalation of coronavirus outbreak. The Guardian. 2nd March 2020. Available at: <https://www.theGuardian.com/business/2020/mar/02/uk-supermarkets-braced-for-stockpiling-if-coronavirus-escalates-panic-buying>
2. Matthews S, Blanchard S, Boyd C. Ocado warns of delivery delays as Brits start hoarding for a coronavirus outbreak. The Daily Mail. 2 March 2020. Available at: <https://www.dailymail.co.uk/news/article-8065099/British-online-supermarket-Ocado-sees-orders-leap-coronavirus-spreads.html>
3. Munbodh E. Supermarkets brace for wave of stockpilers as UK coronavirus fears escalate; Experts have warned that a serious outbreak could result in "panic buying and empty shelves" leaving many supermarkets unable to cope with demand due to the fast-spreading illness. The Daily Mirror. 2nd March 2020. Available at: <https://www.mirror.co.uk/money/supermarkets-brace-wave-stockpilers-coronavirus-21616822>
4. Murray J, Busby M. It's a safety net: across the UK people stock up amid coronavirus fears; The Guardian readers say they are concerned about being forced to self-isolate at home. The Guardian. 2nd March 2020. Available at: <https://www.theGuardian.com/world/2020/mar/02/coronavirus-fears-see-stockpiling-of-food-and-medicines-across-uk>
5. Zeltmann B, Borland H. TAKING STOCK Supermarkets plan to ration food as coronavirus sparks panic buying and stockpiling in the UK. The Sun. 3 March 2020. Available at: <https://www.thesun.co.uk/news/uknews/11085294/supermarkets-plan-coronavirus-brits-stockpile-items/>
6. Benwell S. DON'T PANIC Here's how much food you need to stockpile for two weeks in self-isolation due to coronavirus. The Sun. 4 March 2020. Available at: <https://www.thesun.co.uk/money/11095369/coronavirus-stockpile-guide-how-much/>
7. Barr S. Coronavirus panic-buying: As supermarkets ration items should customers be stockpiling?; 'We understand your concerns but buying more than is needed can sometimes mean that others will be left without' says British Retail Consortium. The Independent. 4 March 2020. Available at: <https://www.independent.co.uk/life-style/food-and-drink/coronavirus-stockpile-emergency-list-food-hand-sanitiser-panic-buying-a9373061.html>
8. No name. Shoppers told 'don't panic' as shelves are cleared. The Metro. 4 March 2020.
9. No name. Stockpiler shop alert. The Sun. 4 March 2020.
10. Poulter S. DON'T PANIC? SHOPPERS CLEAR STORES. The Daily Mail. 4 March 2020.
11. Greep M. Mother with asthma who's bought 150 tins of food and a month's worth of toilet roll amid coronavirus fears is branded 'infuriatingly selfish' by GMB viewers for 'totally unnecessary' stockpiling. The Daily Mail. 5 March 2020. Available at: <https://www.dailymail.co.uk/femail/article-8077657/Mother-bought-150-tins-food-amid-Coronavirus-chaos-branded-selfish-fruit-loop.html>
12. Boyd C. Government accused of risking more panic buying as it advises people to stock up and 'plan ahead' in case they have to self-isolate for two weeks despite pleading with people to stop hoarding. The Daily Mail. March 6 2023. Available at: <https://www.dailymail.co.uk/news/article-8083459/Government-accused-sending-mixed-messages-Brits-told-stock-andself-isolate.html>
13. Saker-Clark H, Munbodh E. Public warned that supermarkets will be well stocked with all supplies despite coronavirus Environment Secretary George Eustice held talks with supermarket and trade body bosses over food supply contingencies on Friday and said he was reassured retailers were taking "all the necessary steps" to keep shelves well-stocked. The Daily Mirror. 6 March 2020. Available at: <https://www.mirror.co.uk/money/public-warned-supermarkets-well-stocked-21647343>
14. Butler S. Supermarkets asked to boost deliveries for coronavirus self-isolation Chains say they are unable to offer many more slots but food shortages remain unlikely. The Guardian. 6 March 2020. Available at: <https://www.theguardian.com/business/2020/mar/06/uk-supermarkets-asked-to-deliver-food-to-people-in-coronavirus-self-isolation>
15. Evans T. Virus CHAOS Rationing hits supermarkets for the first time since WWII due to coronavirus stockpiling. The Sun. 6 March 2020. Available at: <https://www.thesun.co.uk/money/11114222/supermarkets-rationing-wwii-coronavirus/>
16. Merrifield R. Coronavirus: Supermarkets preparing for 'food riots' as panic buying Brits strip shelves Boris Johnson has promised he will keep Britain fed and has reiterated that 'battening down the hatches' and banning social gatherings won't work to stop coronavirus spread. The Daily Mirror. 6 March 2020. Available at: <https://www.mirror.co.uk/news/uk-news/coronavirus-supermarkets-preparing-food-riots-21642224>
17. Poulter S. Supermarkets blast Matt Hancock's 'fanciful, bogus and misleading' claims about mass food deliveries to the sick. The Daily Mail. 7 March 2020. Available at: <https://www.dailymail.co.uk/news/article-8085013/Supermarkets-blast-Matt-Hancocks-fanciful-claims-mass-food-deliveries.html>
18. Dathan M, Crowson I. IT'S SHELF-ISOLATION PANIC-BUYING HITTING SUPERMARKETS Struggle to restock amid frenzy Chaos as Health Sec's contradicted. The Sun. 7 March 2020.
19. Dathan M. PANIC PLANS Government could relax delivery time rules to help supermarkets deal with coronavirus pressure. The Sun. 7 March 2020. Available at: <https://www.thesun.co.uk/news/11118550/relax-delivery-time-rules-ease-coronavirus-pressure-supermarkets/>
20. Street-Porter J. For God's sake, stop all this stockpiling and learn how to be less wasteful instead. The Independent. 7 March 2020.
21. Pidd H. UK supermarkets ration toilet paper to prevent stockpiling Other vanishing items include dried pasta, tinned vegetables, medications and hand gel. The Guardian. 8 March 2020. Available at: <https://www.theguardian.com/world/2020/mar/08/coronavirus-stockpiling-supermarkets-toilet-paper-hand-gel>
22. Knapman H. SELL OUT Supermarket delivery slots sell out over coronavirus stockpiling and shoppers say 'it's worse than Christmas'. The Sun. 8 March 2020. Available at: <https://www.thesun.co.uk/money/11123565/online-delivery-slots-sell-out-coronavirus-christmas/>
23. Gregory A. Coronavirus: Tesco and Waitrose implement rationing due to panic-buying and stockpiling Customers limited to buying no more than five of certain items. The Independent. 8th March 2020. Available at: <https://www.independent.co.uk/news/uk/home-news/uk-supermarkets-coronavirus-stockpiling-rationing-tesco-waitrose-toilet-roll-hand-sanitiser-a9385391.html>
24. Hookam M. SUPERMARKETS TO STOCKPILE IN VIRUS HOTSPOTS. The Daily Mail. 8 March 2020. Available at: <https://www.dailymail.co.uk/news/article-8087227/Supermarkets-stockpile-supplies-coronavirus-hotspots-prevent-food-running-short.html>
25. Maidment J. Government could suspend competition rules and let supermarkets deliver to their rivals' customers in round-the-clock slots if coronavirus crisis hits supply - as panic buyers continue to strip shelves bare. The Daily Mail. 9 March 2020.
26. Bloom D. Coronavirus: Supermarkets to make late-night home deliveries to meet surge in demand Deliveries from big chains like Tesco, Sainsbury's and ASDA are set to be made at previously 'anti-social' hours after the government lifted a set of restrictions. The Daily Mirror. 9 March 2023. Available at: <https://www.mirror.co.uk/news/politics/coronavirus-supermarket-delivery-time-restrictions-21660962>
27. Sales D. VERY LITTTLE HELPS STORES LIMIT STOCKS AMID BUG FEARS Tesco rations pasta & veg to curb panic buys x5 Third Brit dies as virus toll leaps by 67 in one day. The Sun. 9 March 2020.
28. Lewis D. Darren Lewis: Empty shelves from coronavirus panic buying is symptom of me-first society People are losing their minds over the coronavirus as they grab what they can from supermarkets leaving shelves bare of essentials such as toilet roll and pasta. The Daily Mirror. 9 March 2020. Available at: <https://www.mirror.co.uk/news/politics/darren-lewis-empty-shelves-coronavirus-21661932>
29. Hawken L. STOCK TO IT Thrifty mum-of-two reveals she's ALWAYS stockpiled to give family 'security' - and it could save you thousands. The Sun. 9 March 2020. Available at: <https://www.thesun.co.uk/fabulous/11128169/mum-stock-piles-family-security-saved-thousands/>
30. Munbodh E. Coronavirus stockpiling: What each UK supermarket is rationing because of panic-buyers Shelves across the country have been emptied of goods, including toilet paper, after Public Health England urged members of the public to "plan ahead" for if they had to self-isolate for a couple of weeks. The Daily Mirror. 9 March 2020. Available at: <https://www.mirror.co.uk/money/coronavirus-stockpiling-what-each-uk-21658531>
31. Munbodh E. Shoppers being hit with 7-day delays for online grocery orders due to panic-buyers. The Daily Mirror. 9 March 2020. Available at: <https://www.mirror.co.uk/money/shoppers-being-hit-7-day-21659260>
32. Wood Z, Butler S. UK shoppers rush to buy frozen food and freezers amid coronavirus outbreak. The Guardian. 10 March 2020. Available at: <https://www.theguardian.com/business/2020/mar/10/uk-freezer-sales-rise-200-amid-coronavirus-crisis>
33. No name. 1 in 3 admit stockpiling essentials. The Daily Mirror. 10 March 2020.
34. Booth R. Food banks run out of milk and other staples as shoppers panic-buy. The Guardian. 10 March 2023. Available at: <https://www.theguardian.com/world/2020/mar/10/food-banks-run-out-of-milk-and-other-staples-as-shoppers-panic-buy-coronavirus>
35. Winchester L. PRICE FREEZE Where to buy a cheap freezer to help stock up in case of coronavirus self-isolation. The Sun. 10 March 2020. Available at: <https://www.thesun.co.uk/money/11138468/coronavirus-freezer-buy-self-isolate/>
36. Sayid R, Saker-Clark H, Kitching C. Coronavirus outbreak could see army drafted in to deliver food to supermarkets. The Daily Mirror. 11 March 2020. Available at: <https://www.mirror.co.uk/news/uk-news/coronavirus-outbreak-could-see-army-21673145>
37. Ng K. Coronavirus: UK food banks running out of supplies as people stockpile and donations fall. The Independent. 11 March 2020. Available at: <https://www.independent.co.uk/news/uk/home-news/coronavirus-food-bank-stockpile-panic-buying-donations-uk-cases-outbreak-a9393201.html>
38. No name. FROZEN FOOD IS TARGET. The Sun. March 11 2020.
39. No name. FOOD banks are starting to run out of basics amid stockpiling. The Metro. 11 March 2020.
40. Andrews J. Coronavirus: Tesco top boss tells shoppers not to panic as 'there's plenty of food'. The Daily Mirror. 12 March 2020. Available at: <https://www.mirror.co.uk/money/coronavirus-tesco-top-boss-tells-21681991>.
41. Borland H. VIRUS CHAOS: Ocado website goes down and Asda shoppers struggle as supermarkets are hit by coronavirus panic-buying. The Sun. 12 March 2020. Available at: <https://www.thesun.co.uk/money/11156704/ocado-website-asda-supermarkets-coronavirus-panic-buying/>
42. Davidson R. 'I'm ready for the lockdown!' Gemma Collins wears plastic gloves to buy SEVEN packs of toilet roll (and red wine) as she fills TWO trolleys with groceries amid coronavirus outbreak. The Daily Mail. 13 March 2020. Available at: <https://www.dailymail.co.uk/tvshowbiz/article-8109463/Gemma-Collins-wears-plastic-gloves-buy-SEVEN-packs-toilet-roll.html>
43. Matthews L. Coronavirus: Photo shows heartbreaking consequences of panic buying on most vulnerable. The Daily Mirror. 13 March 2020. Available at: <https://www.mirror.co.uk/news/uk-news/coronavirus-photo-shows-heartbreaking-consequences-21686584>
44. No name. Coronavirus: Gemma Collins grabs 7 packs of loo roll and wine in panic shopping spree; Reality TV star Gemma Collins filmed herself filling two trolleys with essentials from toilet paper to red wine in a dash to Marks and Spencers amid fears she'll end up in coronavirus isolation. The Daily Mirror. 13 March 2020. Available at: <https://www.mirror.co.uk/3am/celebrity-news/coronavirus-gemma-collins-grabs-7-21688251>
45. Griffin A. Ocado down: Online shopping app and website break as coronavirus fears lead to panic buying. The Independent. 14 March 2020. Available at: <https://www.independent.co.uk/tech/coronavirus-ocado-down-app-website-stockpiling-food-online-delivery-a9402216.html>
46. Owen G. Supermarket bosses plead for an end to panic buying: Third of shoppers admit stockpiling as stores are stripped bare and left 'looking like a riot zone' as customers scrabble for loo rolls, pasta, and long-life milk. The Daily Mail. 14 March 2020. Available at: <https://www.dailymail.co.uk/news/article-8113007/Panic-buying-rampage-sparks-concern-Number-10-Supermarket-bosses-launch-plea-calm.html>
47. Carr J. Shopper is mugged for his toilet paper in broad daylight outside London shop as supermarkets across Britain have shelves stripped bare amid coronavirus panic. The Daily Mail. 14 March 2020. Available at: <https://www.dailymail.co.uk/news/article-8112481/London-shopper-mugged-toilet-paper-broad-daylight.html>
48. Fiorillo C. Coronavirus: Chaos as shelves 'cleared like a riot' as shoppers mass buy during outbreak. The Daily Mirror. 14 March 2020. Available at: <https://www.mirror.co.uk/news/uk-news/coronavirus-chaos-shelves-cleared-like-21692174>
49. Pochin C. Coronavirus: Jaw-dropping photos of meal prepping and packed freezers amid Covid-19 panic. The Daily Mirror. 14 March 2020. Available at: <https://www.mirror.co.uk/news/world-news/coronavirus-jaw-dropping-photos-meal-21693592>
50. Maidment J. Health Secretary Matt Hancock confirms the elderly WILL be told to stay at home for FOUR MONTHS amid coronavirus crisis - and admits the NHS does not have enough ventilators to cope as death toll hits 35. The Daily Mail. 15 March 2020. Available at: <https://www.dailymail.co.uk/news/article-8113657/Coronavirus-Matt-Hancock-confirms-elderly-told-stay-home-four-months.html>
51. Fiorillo C. Coronavirus: Shoppers queue outside high street shops in frantic bid for toilet rolls. The Daily Mirror. 15 March 2020. Available at: <https://www.mirror.co.uk/news/uk-news/coronavirus-shoppers-queue-outside-high-21696688>
52. Carr J. Supermarkets crackdown on what shoppers can purchase after panic-buyers strip shelves bare with restrictions on items including milk, pasta, and loo roll. The Daily Mail. 15 March 2020. Available at: <https://www.dailymail.co.uk/news/article-8113773/Supermarkets-crackdown-panic-buyers-strip-shelves-bare.html>
53. Griffiths J. AISLE SAY Mum-of-21 Sue Radford insists she WON'T be stockpiling and urges Brits to 'think about the elderly' amid coronavirus. The Sun. 15 March 2020. Available at: <https://www.thesun.co.uk/fabulous/11177903/mum-21-sue-radford-wont-stockpiling-coronavirus-think-about-elderly/>
54. Owen G. PANIC-BUYING RAMPAGE SPARKS CONCERN IN NO 10. The Daily Mail. 15 March 2020. Available at: <https://www.dailymail.co.uk/news/article-8113007/Panic-buying-rampage-sparks-concern-Number-10-Supermarket-bosses-launch-plea-calm.html>
55. King L. Coronavirus: Supermarkets including Aldi beg shoppers for help in stark warning British Retail Consortium members sent a joint letter to consumers urging everyone to work together to ensure there is enough food for the country as panic buying because of the coronavirus outbreak. The Daily Mirror. 15 March 2020. Available at: <https://www.mirror.co.uk/news/uk-news/coronavirus-supermarkets-including-aldi-beg-21697480>
56. King L. Coronavirus: Iceland store opening hour early especially so elderly can get food The heartwarming idea by Iceland in West Belfast Northern Ireland to help elderly customers during the coronavirus outbreak has been applauded on social media including praise from celebrity chef Gino D'Acampo. The Daily Mirror. 15 March 2020. Available at: <https://www.mirror.co.uk/news/uk-news/coronavirus-iceland-store-opening-hour-21698517>
57. Parveen N. Panic buying sweeps stores despite appeal for responsible shopping Empty shelves and complaints of inconsiderate stockpiling as public prepares for self-isolation amid coronavirus crisis. The Guardian. 15 March 2020. Available at: <https://www.theGuardian.com/uk-news/2020/mar/15/panic-buying-sweeps-stores-despite-appeal-for-responsible-shopping>
58. Shaw N. Coronavirus: Items restricted at Asda Tesco Sainsbury's and Morrisons Supermarkets Asda Tesco Sainsbury's Waitrose and Morrisons have all placed restrictions on certain products to stop shoppers stockpiling amid coronavirus fears. The Daily Mirror. 15 March 2020. Available at: <https://www.mirror.co.uk/news/uk-news/coronavirus-items-restricted-asda-teso-21694764>
59. Benwell S. AISLE BE DAMNED Coronavirus - Aldi is restricting shoppers to four items of every product thanks to stockpiling. The Sun. 15 March 2020. Available at: <https://www.thesun.co.uk/money/11176923/aldi-restricting-shoppers-coronavirus-stockpiling/>
60. Pugh R, Symester C. Shopper shares savvy solution to the coronavirus pasta shortage As UK supermarkets continue to battle panic-buying consumers clearing shelves one savvy shopper has spotted an ingenious way to stock up on pasta. The Daily Mirror. 16 March 2020. Available at: <https://www.mirror.co.uk/money/breaking-waitrose-website-crashes-coronavirus-21700850>
61. Twigger M. Coleen Rooney stockpiles for self-isolation amid coronavirus fears with massive shop Wayne Rooney's wife Coleen was snapped leaving a supermarket with mounds of bags as coronavirus fears grown around the world. The Daily Mirror. 16 March 2020. Available at: <https://www.mirror.co.uk/3am/celebrity-news/coleen-rooney-stockpiles-self-isolation-21701766>
62. No name. greed laid bare! STOP STOCKPILING THERE'S ENOUGH FOR EVERYONE SAY SUPERMARKETS. The Metro. 16 March 2020.
63. Winchester L. HELPING HAND Iceland 's special opening hours and coronavirus care packs - what supermarkets are doing to help over-70s. The Sun. 16 March 2020. Available at: <https://www.thesun.co.uk/money/11181020/coronavirus-iceland-supermarket-food-over-70/>
64. Munbodh E. Coronavirus: Waitrose website experiencing issues as Ocado says it's no longer taking new customers Online supermarket Ocado has placed customers in a 'virtual queue' with a wait time of 15 minutes or more as it struggles to cope with a surge of orders due to the coronavirus outbreak. The Daily Mirror. 16 March 2020. Available at: <https://www.mirror.co.uk/money/breaking-waitrose-website-crashes-coronavirus-21700850>
65. Pearson-Jones B. Shopper jokes Ann Summers has VERY rude pasta on offer if 'anyone gets stuck' - after supermarket shelves are stripped of cupboard essentials amid the coronavirus outbreak. The Daily Mail. 16 March 2020. Available at: <https://www.dailymail.co.uk/femail/food/article-8117765/Shoppers-say-theyre-going-buy-Ann-Summers-risque-pasta-supermarkets-sell-out.html>
66. Green J. Wiped out! Mother who stockpiled 18 toilet rolls during coronavirus panic-buying finds her children have thrown them all in the bath. The Daily Mail. 16 March 2020. Available at: <https://www.dailymail.co.uk/news/article-8116839/Mother-stockpiled-18-toilet-rolls-finds-children-thrown-bath.html>
67. Glaze B, Crerar P, Milne O. Coronavirus: 'Stop stockpiling and give to foodbanks' says Welfare Tsar Louise Casey Dame Louise who was appointed by Boris Johnson last month to lead a review into tackling rough sleeping -said it was time for "everybody in our country to step up" and think of those worse off than themselves. The Daily Mirror. 16 March 2020. Available at: <https://www.mirror.co.uk/news/politics/coronavirus-stop-stockpiling-give-foodbanks-21701426>
68. Green J. Supermarket staff report rise in abuse amid coronavirus panic-buying of toilet rolls cleaning products pasta and tinned food despite calls for calm from shoppers. The Daily Mail. 16 March 2020. Available at: <https://www.dailymail.co.uk/news/article-8117527/Trolley-pushing-shoppers-queue-supplies-CostCo-amid-coronavirus-crisis.html>
69. Randall L. Coronavirus: Tesco slashes opening hours at 24-hour stores across the UK Tesco the UK's biggest supermarket chains will reduce its opening hours as Boris Johnson once again warned shoppers about stockpiling -something which has left shelves bare across the UK. The Daily Mirror. 17 March 2020. Available at: <https://www.mirror.co.uk/news/uk-news/breaking-coronavirus-tesco-slash-opening-21709605>
70. Green J, Edmunds C. Toilet roll sales soar by nearly 1000% on Amazon while online retailer shifts double the numbers of aspirin cereal and batteries compared to last year. The Daily Mail. 17 March 2020. Available at: <https://www.dailymail.co.uk/news/article-8120729/Toilet-roll-sales-soar-nearly-1-000-Amazon.html>
71. Green J. Moment stampeding shoppers wrestle each other in panic-buying blitz to grab toilet paper at Home Bargains amid coronavirus chaos. The Daily Mail. 17 March 2020. Available at: <https://www.dailymail.co.uk/news/article-8121921/Stampeding-shoppers-wrestle-panic-buying-crush.html>
72. Leigh-Hurst B. Coronavirus: Doting dad Peter Andre is stockpiling food to keep his kids and wife safe Proud dad-of-four Peter Andre has said he is stockpiling food to feed his family should they need to self-isolate -and admitted his wife Emily is vulnerable to the killer virus. The Daily Mirror. 17 March 2020. Available at: <https://www.mirror.co.uk/3am/celebrity-news/coronavirus-doting-dad-peter-andre-21707012>
73. Osborne S. Shoppers 'queue' for home deliveries as supermarket panic-buying moves online Customers unable to book delivery slots for weeks. The Independent. 17 March 2020. Available at: <https://www.independent.co.uk/news/uk/home-news/coronavirus-shopping-home-deliveries-supermarket-panic-buying-a9406541.html>
74. Greep M. Viewers praise spirit of couple 79 who reveal they're NOT stockpiling and tell GMB they'll spend time watching the birds in their garden amid the coronavirus pandemic. The Daily Mail. 17 March 2020. Available at: <https://www.dailymail.co.uk/femail/article-8120611/GMB-viewers-praise-spirit-elderly-amid-coronavirus-crisis.html>
75. Shadwell T. Coronavirus: McDonald's closes all seating areas at restaurants across UK McDonald's will offer takeaway drive-thru and delivery only during the Covid-19 outbreak and has assured McDelivery orders via Uber Eats and Just Eat will continue -but with 'no contact’. The Daily Mirror. 17 March 2020. Available at: <https://www.mirror.co.uk/news/uk-news/breaking-coronavirus-mcdonalds-closes-seating-21709896>
76. Osborne S. Panic buying moves online. The Independent. 18 March 2020. Available at: <https://www.independent.co.uk/news/uk/home-news/coronavirus-shopping-home-deliveries-supermarket-panic-buying-a9406541.html>
77. Ryan F, Marsh S. Disabled people cut off from vital supplies due to panic buying People with disabilities say coronavirus means they are struggling to get deliveries. The Guardian. 18 March 2020. Available at: <https://www.theguardian.com/world/2020/mar/18/disabled-people-cut-off-from-vital-supplies-due-to-panic-buying-coronavirus>
78. Tweedy J, Enoch N. Ocado closes its website until SATURDAY as online supermarket struggles to keep up with demand from coronavirus stockpilers. The Daily Mail. 18 March 2020. Available at: <https://www.dailymail.co.uk/femail/article-8125961/Frustrated-Ocado-shoppers-reveal-orders-havent-turned-up.html>
79. Donohoe EM. 'This is an essential quarantine supply': Danniella Westbrook shares her VERY extravagant stockpile of pricey champagne as she prepares for self-isolation amid coronavirus panic. The Daily Mail. 18 March 2020. Available at: <https://www.dailymail.co.uk/tvshowbiz/article-8124215/Danniella-Westbrook-shares-extravagant-stockpile-champagne.html>
80. Munbodh E. Coronavirus: Tesco to limit all items to just 3 per person in latest stockpiling ban In a letter to staff chief executive Dave Lewis wrote that all counter services will be closed to focus on stocking shelves from Thursday -with the first hour of the day reserved for the elderly and vulnerable. The Daily Mirror. 18 March 2020. Available at: <https://www.mirror.co.uk/money/breaking-tesco-limit-items-just-21714854>
81. Shadwell T. Sainsbury's introduces coronavirus rationing with some items now two per customer Sainsbury's will introduce new rules limiting how much of each product shoppers can buy launch a special hour for elderly and vulnerable shoppers and boost online deliveries and collection services to cope with coronavirus. The Daily Mirror. 18 March 2020. Available at: <https://www.mirror.co.uk/news/uk-news/sainsburys-introduce-coronavirus-rationing-set-21710398>
82. No name. Stores end loo roll stockpiling. The Daily Mirror. 18 March 2020.
83. Cuthbertson A. Coronavirus: Ocado closes online store due to 'staggering demand'; 'We are fully booked for the next four days' says online supermarket. The Independent. 18 March 2020. Available at: <https://www.independent.co.uk/tech/coronavirus-ocado-online-store-close-virtual-shopping-queue-a9409571.html>
84. Young S. Coronavirus: Sainsbury's introduces rationing which limits customers to three of any product to prevent panic-buying 'We have enough food for everyone' says supermarket boss. The Independent. 18 March 2020. Available at: <https://www.independent.co.uk/life-style/coronavirus-sainsburys-rationing-stockpiling-toilet-roll-soap-uht-milk-a9408086.html>
85. Butler S, Smithers S. UK supermarkets impose tight new rules to prevent hoarding Retailers face struggle to keep shelves stocked during coronavirus crisis. The Guardian. 18 March 2020. Available at: <https://www.theguardian.com/business/2020/mar/18/asda-puts-restrictions-on-shoppers-to-limit-stockpiling-coronavirus>
86. Young S. Coronavirus: Sainsbury's becomes latest supermarket to introduce dedicated shopping hour for elderly and vulnerable 'We have enough food for everyone - if we all just buy what we need for us and our families' says supermarket boss. The Independent. 18 March 2020. Available at: <https://www.independent.co.uk/life-style/coronavirus-sainsburys-elderly-shopping-hours-supermarkets-stockpiling-a9408006.html>
87. Neville S, Randall L. Coronavirus: Elderly man staring at empty Sainsbury's shelves shows panic-buying impact A heartbreaking photo of an elderly man staring at his shopping list in an aisle stripped bare shows the devastating impact Covid-19 panic-buying has on some. The Daily Mirror. 18 March 2020. Available at: <https://www.mirror.co.uk/news/uk-news/coronavirus-elderly-man-staring-empty-21716601>
88. Jones D. SUPERMARKET WEEP STORES BRING IN WW2-STYLE RATIONING; A forlorn woman in Sainsbury's is faced with rows of shelves picked bare by panic-buyers. The Sun. 19 March 2020.
89. Munbodh E. Coronavirus: Lidl's latest rationing guidelines - and what the 4 limit rule applies to The retailer follows the likes of Sainsbury's Asda Morrisons and Waitrose who have all introduced stockpiling bans to help prevent panic-buying during the outbreak. The Daily Mirror. 19 March 2020. Available at: <https://www.mirror.co.uk/money/coronavirus-lidls-latest-rationing-guidelines-21719560>
90. Hiscott G. Supermarkets ration items as riot fears grow. The Daily Mirror. 19 March 2020.
91. Pochin C. Man's heart breaks overhearing elderly shopper ask for eggs - but there aren't any As coronavirus continues to spread across the UK and part of the country faces lockdown shoppers are still wiping out supermarket shelves leaving the most vulnerable in need. The Daily Mirror. 19 March 2020. Available at: <https://www.mirror.co.uk/news/uk-news/mans-heart-breaks-overhearing-elderly-21718182>
92. MacSwan A. Coronavirus: Pensioner 75 left empty-handed issues plea to 'greedy' stockpilers Marilyn Lundy 75 from Urmston, Greater Manchester says a 'disgusting' lack of consideration from panic-buyers has left elderly people like her feeling extremely fearful. The Daily Mirror. 19 March 2020. Available at: <https://www.mirror.co.uk/news/uk-news/coronavirus-pensioner-75-left-empty-21720842>
93. Poulter S. PICTURE THAT SHAMES SELFISH BRITAIN. The Daily Mail. 19 March 2020. Available at: <https://www.dailymail.co.uk/news/article-8126263/Photo-pensioner-clutching-shopping-list-Sainsburys-panic-buyers-ransack-shelves.html>
94. Martin H, Edmunds H. Millions of Sainsbury's, Tesco and Morrisons customers face three-week wait for deliveries as supermarkets are swamped by growing demand for online shops as people self-isolate. The Daily Mail. 19 March 2020. Available at: <https://www.dailymail.co.uk/news/article-8129629/Ocado-sales-soar-stay-home-shoppers-plump-deliveries-amid-pandemic.html>
95. Vine S. SARAH VINE: Panic buyers display the unthinking cruelty of those who care only for themselves. The Daily Mail. 19 March 2020. Available at: <https://www.dailymail.co.uk/debate/article-8132645/SARAH-VINE-Panic-buyers-display-unthinking-cruelty-care-themselves.html>
96. Sulway V. Coronation Street actress Alison King loads car with shopping after stockpiling Coronation Street actress was one of many shoppers this morning who stockpiled their food amid the pandemic concerns. The Daily Mirror. 19 March 2020. Available at: <https://www.mirror.co.uk/3am/celebrity-news/coronation-street-actress-alison-king-21719422>
97. Andrews J. Coronavirus: B&M names the products people are stockpiling most Exactly what people are buying more of has been laid bare by B&M - which just released figures on how much extra it was selling of different items thanks to the coronavirus outbreak. The Daily Mirror. 19 March 2020. Available at: <https://www.mirror.co.uk/money/coronavirus-bm-names-products-people-21718811>
98. Winchester L. STOCK UP Supermarket coronavirus home delivery - how to book a slot to get food delivered to your door. The Sun. 19 March 2020. Available at: <https://www.thesun.co.uk/money/11206595/supermarket-coronavirus-home-delivery-tesco-asda-morrisons-book-rationing>
99. Blewett S, Browne A, Pugh R. Asda, Tesco, Aldi, Sainsbury's and Morrisons opening times and coronavirus stockpiling rules Competition between the UK's biggest supermarkets is being temporarily relaxed so they can collaborate to feed the nation as the shelves are stripped bare during coronavirus outbreak. The Daily Mirror. 19 March 2020. Available at: <https://www.mirror.co.uk/news/uk-news/opening-times-asda-tesco-aldi-21722333>
100. Denton J. Britain's supermarkets are on a war footing against the pandemic: What are their latest steps to help shoppers and how have their shares been affected? The Daily Mail. 19 March 2020. Available at: <https://www.thisismoney.co.uk/money/markets/article-8130535/Britains-supermarkets-war-footing-doing-help-shoppers.html>
101. Laws J. Moment Asda shoppers scuffle over toilet roll and Tesco customer is kicked out in water-stockpiling row as supermarket shelfishness continues. The Daily Mail. 19 March 2020. Available at: <https://www.dailymail.co.uk/news>
102. Walker A. Sainsbury's dedicated shopping hour for vulnerable people 'chaotic and crowded'. The Guardian. 19 March 2020. Available at: <https://www.theguardian.com/world/2020/mar/19/sainsburys-dedicated-shopping-hour-for-vulnerable-people-chaotic-and-crowded>
103. Jones D. COPS & SHOPPERS Police forced to step in after greedy coronavirus panic buyers ransack supermarkets. The Sun. 20 March 2020. Available at: <https://www.thesun.co.uk/news/11221851/police-greedy-ransack-stores-coronavirus/>
104. Grahns A. SO SHELF-ISH Coronavirus panic-buying shoppers spent £60million extra in first week of March. The Sun. 20 March 2020. Available at: <https://www.thesun.co.uk/money/11214803/panic-buying-shoppers-60million-march-coronavirus/>
105. Chapman B. Coronavirus: Supermarkets can now share staff depots and data to help 'feed the nation'. The Independent. 20 March 2020. Available at: <https://www.independent.co.uk/news/business/news/coronavirus-supermarkets-uk-staff-depots-food-a9413146.html>
106. Gallagher S. 'Think before you buy': People with food allergies struggle to get food in wake of coronavirus stockpiling. The Independent. 20 March 2020. Available at: <https://www.independent.co.uk/life-style/food-and-drink/coronavirus-stockpiling-uk-food-allergies-a9414601.html>
107. Pochin C. Coronavirus: Woman in Swiss lockdown shows what UK could have if we stopped panic-buying. The Daily Mirror. 20 March 2020
108. Soteriou S. FOR PETE'S SAKE Peter Andre stunned as daughter Princess insists on going to the shops in her dressing gown and pyjamas. The Sun. 20 March 2020. Available at: <https://www.thesun.co.uk/tvandshowbiz/11215174/peter-andre-princess-shops-dressing-gown/>
109. Elsom J. Sales surge by up to 1237% online for tomato seeds and compost as shoppers start stockpiling more unusual items to help them grow their own food as home office equipment also sell fast as people self-isolate. The Daily Mail. 20 March 2020. Available at: <https://www.dailymail.co.uk/news/article-8135353/Sales-surge-1-237-online-tomato-seeds-compost.html>
110. Mann R. BLOODY IDIOTS Furious husband slams 'horrible' coronavirus panic buyers after Tesco worker wife left in tears after 'worst day ever'. The Sun. 20 March 2020. Available at: <https://www.thesun.co.uk/news/11215372/panic-buyers-coronavirus-tesco-supermarket/>
111. No name. QUEUE STRETCHES THIRD OF A MILE AS BUYERS STOCK UP. The Metro. 20 March 2020.
112. Vonow B. I WAS HELPING OTHERS Retired seaman 79 pictured alone next to empty supermarket shelves slams 'selfish' coronavirus panic-buyers. The Sun. 20 March 2020. Available at: <https://www.thesun.co.uk/news/11216757/retired-seaman-slams-selfish-panic-buyers-coronavirus/>
113. Nikolic I. M&S creates dedicated shopping hours for 'our brilliant NHS workers' and vulnerable customers to help cope with panic-buying stampede. The Daily Mail. 20 March 2020. Available at: <https://www.dailymail.co.uk/news/article-8135215/M-S-creates-dedicated-shopping-hours-brilliant-NHS-workers-vulnerable-customers.html>
114. Saunders E. Coronavirus: Jeremy Vine Show's Andre Walker slams crying nurse who couldn't find food in supermarket. The Daily Mirror. 20 March 2020. Available at: <https://www.mirror.co.uk/tv/tv-news/coronavirus-jeremy-vine-shows-andre-21725083>
115. Martin H. #stophoarding: Furious Britons attack selfish panic-buyers who are emptying shelves of essential items leaving the vulnerable and needy without as supermarkets announce plans to hire thousands of extra workers to cope with soaring demand. The Daily Mail. 20 March 2020. Available at: <https://www.dailymail.co.uk/news/article-8134241/stophoarding-Thousands-furious-Britons-attack-selfish-panic-buyers-emptying-shelves.html>
116. Lavender J. Coronavirus: Woman's genius tip to make own cleaning wipes as shops stripped bare. The Daily Mirror. 20 March 2020. Available at: <https://www.mirror.co.uk/news/uk-news/coronavirus-womans-genius-tip-make-21726156>
117. Tutton C. Coronavirus: Susanna Reid begs for end to stockpiling after 'distressing' NHS nurse video Good Morning Britain host Susanna Reid spoke on the show while self-isolating, urging shop stockpiling to be controlled. The Daily Mirror. 20 March 2020. Available at: <https://www.mirror.co.uk/tv/tv-news/susanna-reid-begs-end-coronavirus-21723705>
118. Riley J. DAY OF THE GOOD… THE MAD… AND THE UGLY THOUSANDS OF PENSIONERS TAKE UP SUPERMARKETS' OFFER OF EXCLUSIVE HOUR TO BEAT PANIC BUYERS. The Metro. 20 March 2020.
119. Truelove S. Coronavirus: Hundreds of shoppers queue around Tesco car park for lockdown supplies Hundreds of shoppers were spotted queuing around the entire car park at Tesco in New Malden, south-west London as stockpiling and panic buying shows no sign of slowing down. The Daily Mirror. 21 March 2020. Available at: <https://www.mirror.co.uk/news/uk-news/coronavirus-hundreds-shoppers-queue-around-21729762>
120. Kindred A. MANIC BUYING Brits have spent £1 BILLION on food in 3 weeks to stockpile at home in coronavirus panic-buying. The Sun. 21 March 2020. Available at: <https://www.thesun.co.uk/news/11224293/coronavirus-panic-buying/>
121. Kitching C. Coronavirus: Brits told 'there is plenty of food' as Government sets up 'war room' Britons have been told to stop panic buying during the coronavirus pandemic and think of NHS workers who find shelves empty after working a long shift on the front line of the fight against Covid-19. The Daily Mirror. 21 March 2020. Available at: <https://www.mirror.co.uk/news/uk-news/breaking-coronavirus-brits-told-there-21730702>
122. Munbodh E. Coronavirus: All the supermarkets offering 'priority hours' for the elderly and NHS staff A number of supermarkets have introduced special shopping hours for pensioners, the vulnerable, NHS workers and paramedics, with the first hour dedicated to them - here's a list of who is participating. The Daily Mirror. 21 March 2020. Available at: <https://www.mirror.co.uk/money/coronavirus-supermarkets-offering-priority-hours-21719925>
123. Truelove S. Coronavirus: NHS boss says panic buyers should be 'ashamed' of themselves Stephen Powis, national medical director at NHS England, said people should be 'ashamed' of themselves for panic buying, and urged people to think of struggling NHS staff. The Daily Mirror. 21 March 2020. Available at: <https://www.mirror.co.uk/news/uk-news/breaking-coronavirus-nhs-boss-says-21730785>
124. Fiorillo C. Coronavirus: Youngest person killed by disease in Britain is man aged just 41 The number of people who have died in the UK after testing positive for Covid-19 reached 233 today after 53 new deaths were confirmed, NHS England said. The Daily Mirror. 21 March 2020. Available at: <https://www.mirror.co.uk/news/uk-news/britains-youngest-coronavirus-victim-man-21731710>
125. Bachelor L. MPs in plea to government over UK's Covid-19 stockpiling Cross-party politicians ask No 10 to safeguard food supplies for NHS and emergency staff Coronavirus. The Guardian. 21 March 2020. Available at: <https://www.theguardian.com/world/2020/mar/21/mps-plea-government-uk-covid-19-stockpiling-coronavirus>
126. Cooney C. STOP BEING SELFISH NHS boss says coronavirus panic-buyers 'should all be ashamed' for leaving medics without food. The Sun. 21 March 2020.
127. Chapman B. Supermarket competition rules suspended amid virus. The Independent. 2020 Mar 21. Available from: <https://www.independent.co.uk/news/business/news/coronavirus-supermarkets-uk-staff-depots-food-a9413146.html>
128. Bullman M, Buchan L. Food bank crisis as £1bn panic-buying spree leaves families facing 'real hunger'. The Independent. 2020 Mar 21. Available from: <https://www.independent.co.uk/news/uk/home-news/coronavirus-foodbanks-stockpiling-uk-panic-buying-supermarkets-food-toilet-paper-a9416326.html>
129. Christodoulou H. 'BE RESPONSIBLE' Brits warned to STOP panic-buying amid coronavirus chaos and told there IS enough food. The Sun. 2020 Mar 21. Available from: <https://www.thesun.co.uk/news/11223898/brits-warned-panic-buying-coronavirus/>
130. Nikolic I, Elsom J. And still they come! Hundreds of shoppers queue all around Tesco car park before 6am waiting for it to open as police step in and supermarkets hire security guards to stop selfish stockpilers amid coronavirus panic. The Daily Mail. 2020 Mar 21. Available from: <https://www.thesun.co.uk/news/11221851/police-greedy-ransack-stores-coronavirus/>
131. Barr S. Coronavirus: Sainsbury's extends dedicated shopping hour to NHS and social care workers; A critical care nurse recently shared a heart-rending video urging shoppers not to stockpile. The Independent. 2020 Mar 21. Available from: <https://www.independent.co.uk/life-style/food-and-drink/coronavirus-supermarket-sainsburys-shopping-hour-elderly-nhs-social-care-workers-covid19-a9415681.html>
132. Woodcock A, Buchan L. Coronavirus: UK 'should be ashamed' after NHS and key workers unable to purchase supplies amid panic buying, says NHS medical chief. The Independent. 2020 Mar 21. Available from: <https://www.independent.co.uk/news/uk/politics/coronavirus-uk-stockpiling-food-panic-buying-nhs-cases-update-today-a9415991.html>
133. Howard H, Martin H. Archbishop of Canterbury urges shoppers to 'please think of others' and stop hoarding after furious Britons attacked selfish panic-buyers who are emptying shelves of essential items. The Daily Mail. 2020 Mar 21. Available from: <https://www.dailymail.co.uk/news/article-8137033/Archbishop-Canterbury-urges-shoppers-think-stop-hoarding.html>
134. Sayid R, Thornton L. Let our NHS heroes eat CORONAVIRUS CRISIS: SELFISH SHOPPERS HIT KEY WORKERS. The Daily Mirror. 2020 Mar 21. Available from: <https://www.mirror.co.uk/news/uk-news/coronavirus-plea-selfish-supermarket-panic-21728742>
135. Jones D. COPS & SHOPPERS POLICE CALLED AS GREEDY RANSACK STORES BOBBIES IN TO STOP VULTURES OAPHOUR OVERRUN BY YOUTH. The Sun. 2020 Mar 21. Available from: <https://www.thesun.co.uk/news/11221851/police-greedy-ransack-stores-coronavirus/>
136. No name. Food banks are vital for the poor - and state inaction and coronavirus stockpiling is putting them under threat Editorial: Further action from the government is required to help thwart stockpilers. It's the best way to ensure there will be more food for everyone, especially those who need it most. The Independent. 21 March 2020. Available at: <https://www.independent.co.uk/independentpremium/voices/food-banks-stockpiling-supermarkets-poverty-freelance-sunak-johnson-a9416321.html>
137. Poulter S. THEY STILL DON'T GET IT. The Daily Mail. 21 March 2020.
138. Pengelly E. Coronavirus: Stranger's act of kindness after stockpiling leaves mum unable to buy food Sophie Gowing was in the Aldershot, Surrey Morrisons when she realised a lack of items on the shelves caused by coronavirus stockpiling had left her unable to afford her usual weekly shop. The Daily Mirror. 21 March 2020. Available at: <https://www.mirror.co.uk/news/uk-news/coronavirus-strangers-act-kindness-after-21732004>
139. Slawson N, Bachelor L. Coronavirus: UK panic-buyers urged to think of frontline workers Government, business and NHS leaders ask people to leave enough supplies for others. The Guardian. 21 March 2020. Available at: <https://www.theguardian.com/world/2020/mar/21/coronavirus-uk-panic-buyers-urged-to-think-of-frontline-workers>
140. Barr S. Dedicated shopping hour at Sainsbury's for NHS staff. The Independent. 22 March 2020. Available at: <https://www.independent.co.uk/life-style/food-and-drink/coronavirus-supermarket-sainsburys-shopping-hour-elderly-nhs-social-care-workers-covid19-a9415681.html>
141. Howes S. SO WHY IS IT ONLY THE BRITS WHO STOCKPILE? The Mail on Sunday. 22 March 2020.
142. Craven N. AND EVEN IF STOCKPILING FIZZLES OUT, STORE CHIEFS EXPECT PROFITS SURGE TO RUN ON FOR MONTHS. The Daily Mail. 22 March 2020.
143. Wooding D, Jones D, Pollard C. £1BN SHOP MUST STOP FULL SCALE OF STOCKPILING REVEALED Officials blast hoarders 'Should be ashamed' 6am queues at Tesco NHS workers go unfed. The Sun. 22 March 2020.
144. Cordon G. 'You should all be ashamed of yourselves' PANIC BUYERS CRITICISED AS NHS STAFF LEFT WITHOUT FOOD. The Sunday Sun. 22 March 2020.
145. Harrison L. Kathy Burke responds to backlash over joke about 'stockpiling biscuits' amid coronavirus outbreak Comedian hit back at those who took offence to her 'mild, silly tweet.' The Independent. 23 March 2020. Available at: <https://www.independent.co.uk/arts-entertainment/tv/news/coronavirus-kathy-burke-stockpiling-biscuits-joke-twitter-a9417951.html>
146. O' Grady S. If selfish Britons can't keep their distance, the government will have to scare them into saving lives Selfish behaviour, among other things, has got us to where we are now and we are not going to turn human nature upside down. The Independent. 23 March 2020. Available at: <https://www.independent.co.uk/voices/coronavirus-social-distancing-supermarkets-parks-boris-johnson-a9418666.html>
147. Holland P. Coronavirus: Stockpilers shout abuse at female Tesco worker -now her partner hits back Tesco employee Angela Breathet returned home from work in tears after being verbally abused by shoppers who continue to ignore COVID-19 advice to stop stockpiling essential items. The Daily Mirror. 23 March 2020. Available at: <https://www.mirror.co.uk/news/weird-news/coronavirus-stockpilers-shout-abuse-female-21739268>
148. Kent SA. Coronavirus: Christine McGuinness begs stockpiling shoppers to think of her autistic kids. The Daily Mirror. 23 March 2020. Available at: <https://www.mirror.co.uk/3am/celebrity-news/coronavirus-christine-mcguinness-begs-stockpiling-21742701>
149. Elsom J. Tesco reduces skimmed milk stock, removes single-pint cartons and scraps multi-buy offers in bid to ensure supply of essential goods after coronavirus panic-buyers strip shelves. The Daily Mail. 24 March 2020. Available at: <https://www.dailymail.co.uk/news/article-8147701/Tesco-stops-selling-skimmed-milk-scraps-multi-buy-offers.html>
150. Lockett J. LINE SPACING Shoppers line up with proper coronavirus 'social distancing' as they queue around car parks to get into supermarkets. The Sun. 25 March 2020. Available at: <https://www.thesun.co.uk/news/11254413/shoppers-social-distance-supermarkets-queues/>
151. Farmer C. 'The world is a hateful place': Michelle Heaton is slammed for large food shop amid coronavirus pandemic... as she hits back by revealing the haul is for her family AND two high-risk pals. The Daily Mail. 25 March 2020. Available at: <https://www.dailymail.co.uk/tvshowbiz/article-8151489/Michelle-Heaton-lashes-cruel-trolls-criticised-large-food-shop.html>
152. Whitbread L. Which supermarkets have delivery slots available? The Independent. 25 March 2020.
153. Platt-Leonard J. 20 pledges for 2020: How labelling and storing your food can help to avoid coronavirus panic buying. The Independent. 25 March 2020. Available at: <https://www.independent.co.uk/20-pledges/coronavirus-food-sustainability-storage-label-freezer-items-supermarket-a9423531.html>
154. Boyle D, Gordon A. Young people are urged to stay away from supermarkets and make food last longer as it emerges Brits have stockpiled £1bn worth of goods in their cupboards - as stores erect barriers to keep crowds at bay and online shoppers are left in virtual queues. The Daily Mail. 26 March 2020. Available at: <https://www.dailymail.co.uk/news/article-8154905/Tesco-asks-young-people-stop-ordering-food-online-shop-store.html>
155. Boyd M. Coronavirus: UK faces WWII rationing if stockpiling doesn't stop warns expert. The Daily Mirror. 26 March 2020. Available at: <https://www.mirror.co.uk/news/uk-news/coronavirus-uk-faces-wwii-rationing-21760398>
156. Wood Z. 'Nobody will starve,' says Ocado chair, urging Britons to stop stockpiling. The Guardian. 25 March 2020. Available at: <https://www.theguardian.com/world/2020/mar/26/nobody-will-starve-says-ocado-chair-urging-britons-to-stop-stockpiling>
157. Osborne S. How much food do you need to eat?: Ocado boss pleads with UK public to stop stockpiling; 'Nobody will starve,' Lord Rose says. The Independent. 26 March 2020. Available at: <https://www.independent.co.uk/news/uk/home-news/coronavirus-supermarket-ocado-sainsburys-tesco-asda-lidl-stockpile-groceries-a9427056.html>
158. Osborne S. Coronavirus: Iceland boss urges Britons to ignore Boris Johnson's advice on shopping. The Independent. 27 March 2020. Available at: <https://www.independent.co.uk/news/uk/home-news/coronavirus-shopping-delivery-iceland-online-elderly-vulnerable-boris-johnson-a9429831.html>
159. Layton J. Outrage as bins overflow with Brits' out-of-date food bought in coronavirus panic. The Daily Mirror. 28 March 2020. Available at: <https://www.mirror.co.uk/news/uk-news/coronavirus-outrage-bins-pictured-filled-21773469>
160. Steed L. SHOPPING IN 2020 Shoppers in protective gear, screens put up in supermarkets and Tesco limits key items to one per person. The Sun. 29 March 2020. Available at: <https://www.thesun.co.uk/money/11279771/shoppers-protective-gear-screens-supermarkets-tesco-limits/>
161. Wood Z. Supermarkets ready for a new week of rising to the virus's challenge Supermarkets have won praise for their response to the crisis. The next issue may be keeping staff healthy. The Guardian. 29 March 2020. Available at: <https://www.theguardian.com/business/2020/mar/29/supermarkets-ready-new-week-virus-challenge>
162. Saunders E. Shop worker documents 'horrific' scenes as crazed shoppers abuse her for not putting out stock fast enough The coronavirus pandemic has brought out the best -and worst -in some people, as this shop assistant opened up about the most 'shocking and terrible' behaviour she's witnessed in her store in nearly 20 years. The Daily Mirror. 29 March 2020. Available at: <https://www.mirror.co.uk/tv/tv-news/shop-worker-documents-horrific-scenes-21774346>
163. O'Leary A. Panic buyers slammed for dumping perfectly good food amid fears of rat invasion Piles of uneaten foodstuffs have begun piling up as people who greedily filled their trolleys and hoarded goods amid coronavirus fears -often depriving the most vulnerable in our communities of basic necessities. The Daily Mirror. 30 March 2020. Available at: <https://www.mirror.co.uk/news/uk-news/panic-buyers-who-stockpiled-goods-21782809>
164. Yeatman D. NEEDLESS WHAT A WASTE! NOW PANIC BUYERS CHUCK OUT-OF-DATE FOOD. The Metro. 30 March 2020.
165. Hawkins J. Giant rats 'could invade streets and homes' as COVID-19 stockpilers dump spoiled food Experts say rodents will sweep the country because of stockpilers who are dumping food, which could attract rats of more than a foot long and spread illnesses through their urine. The Daily Mirror. March 30 2020. Available at: <https://www.mirror.co.uk/news/uk-news/giant-rats-could-invade-streets-21779704>
166. Borland S. SUPER market March BIGGEST month on record for grocery sales with households spending extra £63 in supermarkets, according to Kantar. The Sun. 31 March 2020. Available at: <https://www.thesun.co.uk/money/11292159/march-biggest-month-record-grocery-sales/>
167. Smithers R. Britons made 80m extra grocery shops in less than a month Between 24 February and 21 March, UK shoppers spent an extra £2bn on food and drink. The Guardian. 31 March 2020. Available at: <https://www.theguardian.com/business/2020/mar/31/coronavirus-britons-made-80m-extra-grocery-shops-in-less-than-a-month>
168. Wood Z. UK supermarkets ease coronavirus shopping restrictions Aldi, Lidl, Morrisons and Waitrose relax quantity curbs after decline in footfall. The Guardian. 31 March 2020. Available at: <https://www.theguardian.com/business/2020/mar/31/uk-supermarkets-ease-coronavirus-shopping-restrictions>
169. Munbodh E. Coronavirus panic-buying makes March busiest month on record -even beating Christmas The average household has increased its spending by £62.92 over the last four weeks, with shoppers in London, where the coronavirus hit first, spent a quarter more than they usually do. The Daily Mirror. 31 March 2020. Available at: <https://www.mirror.co.uk/money/march-busiest-month-record-supermarkets-21785401>
170. Bradbury S. What supermarkets still have delivery slots? Rules for Morrisons, Sainsbury's and more Some supermarkets including Waitrose and Iceland have been inundated with home deliveries during the coronavirus pandemic and we have all the information you need right here. The Daily Mirror. 31 March 2020. Available at: <https://www.mirror.co.uk/money/shopping-deals/what-supermarkets-still-delivery-slots-21785214>
171. Hawken A. WHAT A WASTE Fury as stockpilers throw away mountains of food bought in panic-buyer supermarket frenzy. The Sun. 31 March 2020. Available at: <https://www.thesun.co.uk/news/11293013/fury-stockpilers-throw-away-food-coronavirus/>
172. Sayid R. Supermarkets have biggest month ever with huge 20% coronavirus sales spike Supermarkets have had a bigger four weeks in terms of sales than those preceding Christmas, with Aldi, Morrisons, Lidl, Tesco and Waitrose all seeing big spikes in customers. The Daily Mirror. 31 March 2020. Available at: <https://www.mirror.co.uk/news/uk-news/supermarkets-biggest-month-ever-huge-21790037>
173. Campbell M. I've worked to curb hunger for years - watching food waste from stockpiling mount up is heartbreaking Help The Hungry: Images of discarded food are an insult to anyone experiencing poverty right now - and social distancing means invaluable provisions like breakfast clubs cannot be facilitated. The Daily Mirror. 1 April 2020. Available at: <https://www.independent.co.uk/voices/homeless-stockpiling-help-hungry-coronavirus-food-banks-a9440231.html>
174. **Sayid R.** STORE CRAZY CORONAVIRUS CRISIS: SHOPPING CHAOS REVEALED» £11bn spent panic buying in one month 'Shop once a week' Tory slapped down Shops end rationing on many goods. The Daily Mirror. 2020 Apr 1.
175. **Kaur M.** Supermarkets including Asda, Aldi and Morrisons lift some restrictions on items Several supermarkets have eased buying restrictions which were recently put in place to prevent stockpiling. Here's where shoppers will only face limits on a handful of items. The Daily Mirror. 2020 Apr 1. Available from: <https://www.mirror.co.uk/money/shopping-deals/supermarkets-including-asda-aldi-morrisons-21791611>.
176. **Street S.** SPECIAL DELIVERY Iceland home delivery - what time are the slots released online?. The Sun. 2020 Apr 2. Available from: <https://www.thesun.co.uk/money/11304513/iceland-home-delivery-slots-opening-time-coronavirus-uk-lockdown/>.
177. **Einstein A.** FIRED UP The coronavirus stockpilers who use BB guns & chainsaws to protect their huge hauls & even keep them under lock & key. The Sun. 2020 Apr 3. Available from: <https://www.thesun.co.uk/fabulous/11313927/coronavirus-stockpilers-bb-gun-chainsaw-huge-hauls-protect-lock/>.
178. **Wilson B.** Off our trolleys: what stockpiling in the coronavirus crisis reveals about us. The Guardian. 2020 Apr 3. Available from: <https://www.theguardian.com/news/2020/apr/03/off-our-trolleys-what-stockpiling-in-the-coronavirus-crisis-reveals-about-us>.
179. **Munbodh E.** Coronavirus: Aldi lifts rationing limits and introduces new priority hours from today Aldi has lifted purchasing restrictions on all products from Monday, except hand wash, which is limited to four per customer, shower gel, limited to two per customer, and UHT (long-life) milk, limited to two per customer. The Daily Mirror. 2020 Apr 4. Available from: <https://www.mirror.co.uk/money/aldi-lifts-rationing-limits-introduces-21820037>.
180. **Munbodh E.** Coronavirus: Sainsbury 's changes its policy on Easter eggs and dozens more items Supermarket giant Sainsbury 's has said it's relaxing rules from Monday, including rationing limits on key lines such as milk and canned foods. Priority hours will however remain the same. The Daily Mirror. 2020 Apr 4.
181. **Nisbett M, Andrews J.** Supermarket Easter egg buying rules this week as shops make changes Here are the rules for buying Easter eggs at Sainsbury's, Aldi, Asda and more this week -as well as when the shops will be open over the bank holiday weekend. The Daily Mirror. 2020 Apr 7. Available from: <https://www.mirror.co.uk/money/supermarket-easter-egg-buying-rules-21829827>.
182. Knight K. MORAL MAZE Takeaways are OK - but call the cops on those park covidiots! Our expert answers your ethical coronavirus dilemmas. The Sun. 7 April 2020. Available at: <https://www.thesun.co.uk/news/11309960/coronavirus-moral-maze-dilemmas-older-sick-die-ordering-online/>.
183. Borland S. SUPERMARKET SWEEP 9 supermarket tricks from getting delivery slots to when is the best time to visit. The Sun. 8 April 2020.
184. Boles T. Can do at Tesco SALES RISE 30 PER CENT l45,000 NEW STAFF HIRED. The Sun. 9 April 2023.
185. Munbodh E. Aldi's new opening hours for shoppers come into effect today German discounter Aldi has become the latest to lift restrictions and make changes to opening hours as families across the UK prepare for several more weeks in lockdown. The Daily Mirror. 9 April 2020. Available at: <https://www.mirror.co.uk/money/breaking-aldi-extends-supermarket-opening-21840063>.
186. Murphy-Bates S. Middle classes in south of England were to blame for stockpiling during coronavirus crisis, Tesco boss claims as panic buying sent sales soaring by a third. The Daily Mail. 9 April 2020. Available at: <https://www.dailymail.co.uk/news/article-8203689/Middle-classes-south-England-blame-stockpiling-coronavirus-crisis.html>.
187. Einstein A. BINGE BUY I stockpiled dozens of eggs, fruit & sauces but had to bin it all - I don't care I'm doing the vulnerable out of food. The Sun. 9 April 2020. Available at: <https://www.thesun.co.uk/fabulous/11347343/stockpile-food-bin-all-no-regrets/>.
188. O'Leary A. Stockpiling mum who splurged £250 forced to bin food and even toilet roll that 'got wet'. The Daily Mirror. 10 April 2020. Available at: <https://www.mirror.co.uk/news/uk-news/stockpiling-mum-who-splurged-250-21839254>.
189. Sunderland R. WE HAVE CHANGED MORE IN A FEW WEEKS THAN IN TEN YEARS. The Daily Mail. 14 April 2020. Available at: <https://www.thisismoney.co.uk/money/markets/article-8215263/We-changed-weeks-TEN-years-says-Tesco-boss.html>.
190. Munbodh E. Many supermarket prices soaring during lockdown - but some goods are now cheaper. The Daily Mirror. 17 April 2020. Available at: <https://www.mirror.co.uk/money/coronavirus-supermarket-items-soared-price-21882452>
191. Knapman H. PRICE IS RIGHT Cost of weekly supermarket shop jumps by up to 10% after coronavirus panic buying sees discounts pulled. The Sun. 18 April 2020. Available at: <https://www.mirror.co.uk/money/coronavirus-supermarket-items-soared-price-21882452>
192. Shaw N, Fiorillo C. Lockdown driving rats into our homes as pest control reports spike in activity. The Daily Mirror. 19 April 2020. Available at: <https://www.mirror.co.uk/news/uk-news/coronavirus-lockdown-driving-rats-homes-21890794>
193. Rogers J. RAT RACE Rats invading homes and turning into 'cannibals' due to coronavirus starvation after restaurants shut. The Sun. 19 April 2020. Available at: <https://www.thesun.co.uk/news/11431202/coronavirus-lockdown-rats-homes-restaurants-shut/>
194. Bass C. PILE UP Proud mum shows off her newly-organised pantry but sparks fierce debate about whether she's stockpiling supplies. The Sun. 20 April 2020. Available at: <https://www.thesun.co.uk/fabulous/food/11434464/proud-mum-organised-pantry-fierce-debate-about-stockpiling-supplies/>
195. Butler S. UK sales of baking goods and alcohol rise while makeup and plants fall. The Guardian. 20 April 2020. Available at: <https://www.theguardian.com/business/2020/apr/20/uk-sales-of-baking-goods-and-alcohol-rise-while-makeup-and-plants-fall>
196. O'Carroll L, Brooks L. Corner shops describe life under lockdown as they stay open for their community. The Guardian. 22 April 2020. Available at: <https://www.theguardian.com/business/2020/apr/22/corner-shops-describe-life-under-lockdown-as-they-stay-open-for-their-community>
197. Matthews L. Mum accused of stockpiling after proudly posting photos of her food cupboard. The Daily Mirror. 22 April 2020. Available at: <https://www.mirror.co.uk/lifestyle/mum-accused-stockpiling-after-proudly-21906265>
198. Inman P. UK lockdown sends retail sales to record low, but alcohol sales rise. The Guardian. 24 April 2020. Available at: <https://www.theguardian.com/business/2020/apr/24/uk-lockdown-sends-retail-sales-to-record-low-but-alcohol-sales-rise>
199. Partridge J. Sainsbury's top man steps down just as the panic-buying revenues roll in. The Observer. 26 April 2020. Available at: <https://www.theguardian.com/business/2020/apr/25/sainsburys-man-at-top-steps-down-panic-buying-revenues-mike-coupe>
200. Stubbings D. Tesco, Aldi, Asda and Morrisons' latest lockdown shopping rules explained Rules for shoppers at supermarkets have frequently changed during the coronavirus pandemic. Here's a round-up of the latest rules from the biggest chains including Tesco, Aldi and Asda. The Daily Mirror. 28 April 2020. Available at: <https://www.mirror.co.uk/money/tesco-aldi-asda-morrisons-latest-21937811>.
201. Munbodh E. Sainsbury's boss warns coronavirus queues and restrictions will last until September Chief executive Mike Coupe, who is to step down in June, said the grocer expects lockdown restrictions to have eased by the end of June but that business disruption will continue until autumn. The Daily Mirror. 30 April 2020. Available at: <https://www.mirror.co.uk/money/sainsburys-boss-warns-coronavirus-supermarket-21953786>.
202. Butler S. Sainsbury's boss warns coronavirus queues and restrictions will last until September Chief executive Mike Coupe, who is to step down in June, said the grocer expects lockdown restrictions to have eased by the end of June but that business disruption will continue until autumn. The Guardian. 30 April 2020. Available at: <https://www.theguardian.com/business/2020/apr/30/sainsburys-boss-warns-coronavirus-disruption-will-last-until-mid-september>.
203. Davis P. Coronavirus lockdown is plunging people with eating disorders into deeper crisis EXCLUSIVE: Eating disorder sufferers told Mirror Online that lack of routine, fears over food stockpiling and reduced access to mental health services has left many with eating disorders such as anorexia and bulimia struggling to cope. The Daily Mirror. 7 May 2020. Available at: <https://www.mirror.co.uk/news/uk-news/coronavirus-lockdown-plunging-people-eating-21984150>.
204. Manning D, Kitching C. Coronavirus: Latest trolley limits for Tesco, Aldi, Asda and Sainsbury's shoppers There are generally good levels of stock at UK supermarkets but Tesco, Sainsbury's, Asda, Marks & Spencer, Aldi and Waitrose are among those who still have restrictions on some of the most popular items. The Daily Mirror. 16 May 2023. Available at: <https://www.mirror.co.uk/news/uk-news/coronavirus-latest-trolley-limits-tesco-22037154>.
205. Tapsfield J. Retail sales plummeted by a FIFTH in April as Britons stopped buying fuel and clothing amid coronavirus lockdown - but purchases of alcohol were UP. The Daily Mail. 22 May 2020. Available at: <https://www.dailymail.co.uk/news/article-8347151/Retail-sales-plummeted-FIFTH-April-amid-coronavirus-lockdown.html>.
206. Vaghela K. Crack in fashion! Tesco stocks white eggs for the first time in more than 40 years after huge rise in demand. The Daily Mail. 25 May 2020. Available at: <https://www.dailymail.co.uk/news/article-8354121/Tesco-stocks-white-eggs-time-40-years-meet-unprecedented-demand.html>.
207. Smithers R. White shelled eggs are selling in Tesco for the first time in 40 years Coronavirus lockdown gives new lease of life to variety used mainly by restaurants. The Guardian. 25 May 2020. Available at: <https://www.theguardian.com/business/2020/may/25/white-shelled-eggs-are-selling-in-tesco-for-the-first-time-in-40-years>.
208. O'Sullivan K. Most dangerous part of the supermarket - with one key thing you should look out for The supermarkets coronavirus secrets have been exposed in Keeping Britain Fed -from why eggs are now white to reason breweries have ditched making kegs for hand sanitiser. The Daily Mirror. 17 June 2020. Available at: <https://www.mirror.co.uk/tv/tv-news/most-dangerous-part-supermarket-lazy-22206836>.
209. Smithers R. Food waste increases in UK as coronavirus restrictions ease Levels are still below those recorded before lockdown, says government advisory body. The Guardian. 29 July 2020. Available at: <https://www.theguardian.com/environment/2020/jul/29/food-waste-increases-in-uk-as-coronavirus-restrictions-ease>.
