# Supplementary material for: The portrayal of panic-buying and stockpiling in English newspapers during Covid, a mixed-method content analysis
Source: PLoS One. 2025 Feb 12;20(2):e0315142. doi: 10.1371/journal.pone.0315142 (PMC11819479; doi:10.1371/journal.pone.0315142)
Supplement: Attachment — Submitted filename: Response to Reviewers.docx [file pone.0315142.s004.docx]

Dayna Brackley & Rebecca Wells

Centre for Food Policy,

City St George’s,

University of London,

Northampton Square,

London,

EC1V 0HB

5^th^ November 2024

PLOS One

PLOS Nine Hills Road,

Cambridge,

CB2 1GE

Dear Peer Reviewers,

We would like to express our sincere thanks for your thoughtful and detailed feedback on our manuscript submitted to *PLOS ONE*. Your comments and suggestions have been extremely helpful in refining and improving the clarity and quality of our work.

We have carefully considered each point raised and made the appropriate revisions. To assist with the review process, we have included a table that outlines your comments along with our responses and the corresponding revisions or rebuttals.

Your input has greatly contributed to enhancing the manuscript, and we are grateful for the time and effort you put into your reviews. We look forward to any further feedback you may have.

Best regards,

Dayna Brackley
Rebecca Wells

| **Reviewer** | **Reviewer Feedback** | **Author Response** |
| --- | --- | --- |
| Editor | Please ensure that your manuscript meets PLOS ONE's style requirements, including those for **file naming**. | Thank you for this comment. The author has gone through line by line to check styling and can confirm:  Font size: 12, double-spaced, 3 heading levels, page numbers and line numbers, abbreviations defined on first use if used, references in Vancouver style, correct manuscript organisation, figure captions inserted immediately after first paragraph in which cited, tables after the first paragraph in which cited. The author is unclear as to what file name is not meeting the guidance - the Supporting Files are named appropriately, Figures listed as Fig1/Fig2. The authors will happily adjust if more guidance could possibly be provided. |
| Editor | Please ensure that your manuscript meets PLOS ONE's style requirements, including those for **author affiliations**. | We have also updated the affiliation for Dayna Brackley to be at the Centre for Food policy, according to the guidance " their affiliation at the time of the study". Dayna as a former student no longer has access to a City.ac.uk email, and has therefore listed her current email. |
| Editor | **Include additional papers**: Sherman, C. E., Arthur, D., & Thomas, J. (2021). Panic buying or preparedness? The effect of information, anxiety and resilience on stockpiling by Muslim consumers during the COVID-19 pandemic. Journal of Islamic Marketing, 12(3), 479-497. Lehberger, M., Kleih, A. K., & Sparke, K. (2021). Panic buying in times of coronavirus (COVID-19): Extending the theory of planned behavior to understand the stockpiling of nonperishable food in Germany. Appetite, 161, 105118. Gong, J., Said, F., Ting, H. et al. Do Privacy Stress and Brand Trust still Matter? Implications on Continuous Online Purchasing Intention in China. Curr Psychol 42, 15515–15527 (2023). https://doi.org/10.1007/s12144-022-02857-x | Thank you for these most useful paper recommendations. You will see that the authors have included references to Sherman *et al* and Lehberger *et al* on page 6. These were useful additions. Privacy stress and online shopping in China in *Gong* et al, did not have relevant information to the study, and was therefore not included. |
| Editor | From the portrayal of panic buying on newspaper, we shall also talk about how to be responsible or make it a responsible reporting, marketing , and benefit the stakeholder. I hope authors could talk a bit about responsibility for both journalists and marketers. One paper for your to refer: Ting, H., Gong, J., Cheah, J.H.(J). and Chan, K. (2024), "Editorial: The infodemic, young consumers and responsible stakeholdership", Young Consumers, Vol. 25 No. 4, pp. 421-424. https://doi.org/10.1108/YC-04-2024-2059 | Thank you for this comment. We have included the useful reference to the infodemic on page 5. We hope that Table 7, on page 42/43 which offers a panic-buying reporting framework for National Press speaks to responsible journalism. Marketing was out of scope for this paper. |
| Editor | Lastly, please double-check you language and i found some minor language issues and hope you can check carefully before submitting back to the journal. | The authors have checked the manuscript for minor language and grammar issues and made adjustments. |
| Reviewer 2 | Regarding language and grammar, a thorough review of the text might be beneficial. | As above |
| Reviewer 2 | You might want to include additional bibliography. | We have extended the bibliography including the suggested papers from the editor, we thank the reviewer for this comment. |
| Reviewer 1 | I encourage the authors to ensure that the study maintains a clear academic focus, avoiding any potential misinterpretations or politicisation of the subject | The author has identified four potential areas where there could be an interpretation of politicisation/misinterpretation:  1) criticism of the government, 2) media sensationalism, 3) supermarkets as heroes and individuals as villains and 4) social inequity and panic buying.  1) For the criticism of the government, the authors think it is clear that this criticism is from the Environment, Food and Rural Affairs Committee, rather than the authors themselves.  2) For media sensationalism, the authors have adjusted language on page 6 in light of the reviewer comment. The authors believe that they clearly defined sensationalism, and gave robust examples of where this was found across newspapers during the study.  3) For individuals as villains, the author has adjusted language removing the term demonise/demonisation, however the study very much indicated that individuals were blamed in language terms and Table 4 gives specific examples of this. For supermarkets as heroes, the author adjusted language, however the study clearly showed supermarkets spoken about in heroic terms, so the authors believe this language is justified.  4) For social inequity, the author has adjusted the language in this section, in light of the reviewer comments, but argue that the evidence base is clear that panic buying contributed to social inequity. |
| Reviewer 1 | **Abstract:** Clarify key terms and scope: I think the abstract should briefly define key concepts such as "sensationalised language" and explain the criteria used for coding articles as "negative" or "positive." This will help readers understand the methodology more clearly. | Thank you for this comment. We direct the reviewer to Table 2 which has a definition framework for positive, negative, neutral, mixed and sensationalism. We also give examples of sensationalism in Table 2 to fully explain what is meant using live examples from newspaper articles in the study. This is within the methodology section, we are not able to include in the Abstract due to the short word limit. |
| Reviewer 1 | **Abstract:** Strengthen the connection between findings and implications: After mentioning the six key themes and conclusions, the abstract should explicitly connect these insights to the potential impact on policymaking and crisis communication, offering a more direct conclusion about the relevance of the research for future crises. | Thank you for this useful comment - the author has re-ordered the abstract, and in particular re-ordered the last paragraph to explicitly connect the insights to the policy and communication implications. |
| Reviewer 1 | **Abstract:** Consolidate the abstract into a single cohesive paragraph: Merging the abstract into one well-structured paragraph will enhance its flow and readability, ensuring that the study's background, objectives, methodology, key findings, and implications are presented in a more concise and integrated manner, avoiding unnecessary fragmentation. | Thank you for this useful comment - the author has merged the abstract into a single paragraph, and made further changes as per the reviewers other comment on strengthening the connection between findings and implications. |
| Reviewer 1 | **Keywords:** Enhance Searchability: Please list your keywords to enhance the searchability of this study. | This is a welcome, useful suggestion, we have listed out a section of Keywords after the abstract. |
| Reviewer 1 | **Introduction:** Clarify the research gap earlier: I hope you can introduce the lack of media content analysis on Covid panic-buying in the UK press earlier in the introduction. This will help readers understand the study's unique contribution from the start and emphasise the research's relevance in filling this gap. | As we have re-ordered the introduction, the authors believe that this research gap is contextually best placed near the research questions and hope that the re-ordered introduction now is fit for purpose. |
| Reviewer 1 | **Introduction:** Streamline the government response section: The introduction contains many details on the government's actions and criticisms. I suggest the you consider summarising this more succinctly to avoid overwhelming the reader while still providing context for the role of media in public perception and government accountability. | The government information has been streamlined and reduced in word count. |
| Reviewer 1 | **Introduction**:Enhance the flow between media and panic-buying discussions: To strengthen the argument, you need to enhance the connection between media portrayal and panic-buying behaviour could be more clearly emphasized. Transition more smoothly between discussing the media’s role and its impact on public behaviour to keep the focus clear and coherent. | The author has revised the introduction to enhance the flow between media and panic buying and significantly re-ordered. |
| Reviewer 2 | **The introduction** is rich in information. Perhaps you could consider dividing it into subsections (e.g., causes of panic-buying, government responses, role of the media) to enhance readability and structure. While the research background is well-described, you might want to articulate the specific aims, methods, and supporting theories more clearly. It could be beneficial to explicitly state the research purpose, questions, and significance at the end of the Introduction | Thank you for this comment, we have divided the introduction into subsections. We have now updated the research aims section, and as suggested stated the purpose, research questions and significance at the end of the introduction. |
| Reviewer 1 | **Research Questions**: I strongly recommend including research questions at the end of the Introduction to better guide readers and clarify the focus of your study. | As above, this is useful and we have now included the research questions. |
| Reviewer 2 | Regarding the **theoretical framework**, you might consider elaborating on the theories underpinning your study. For instance, you could clarify how the Agenda-setting theory or framing theory guides your research, and justify their application. | You will see that the research aims and contributions section now clarifies the two theories and justifies their usage, thank you for this comment. |
| Reviewer 1 | **Literature Review:** Why don’t you write a literature review for this work? I think separating the introduction from the literature review part is necessary. | We appreciate the feedback suggesting that a literature review be incorporated into the introduction section and we have re-ordered it and included a literature review and incorporated the other introduction feedback. |
| Reviewer 1 | **Method:** Clarify the rationale for newspaper selection: While the selection of six popular newspapers is explained, it would be helpful to clarify why these specific titles were chosen about their readership and political stance and how these factors might influence the portrayal of panic-buying, providing a more substantial justification for the sample. | Thank you for these comments, you will see this has been incorporated into the method section. |
| Reviewer 1 | **Method:** Simplify technical terms for broader readability: Terms like "manifest" and "latent content" could be briefly simplified or explained in more accessible language to ensure a wider audience understands the methodology without needing specialised knowledge of content analysis. | Thank you, we have updated the language to ensure it is simplified. |
| Reviewer 1 | **Method:** Expand on Nexis's limitations: The paper briefly mentions Nexis's limitation of not providing visual content. Why not expand on how this limitation might have impacted the data collection process or how the scope of visual analysis would add transparency to the methodology? | The author has updated this section on Nexis limitations to expand on the limitation and how it impacted data collection. |
| Reviewer 2 | In the **Methodology** section, you might find it helpful to provide more detailed descriptions of content analysis methods and visual content analysis techniques. Perhaps you could strengthen the explanation of how visuals are systematically coded and how these visual elements link to your research concerns. Including specific examples of the coding and analysis process could enhance clarity and replicability. | The methodology has now been updated following this useful comment. The authors have included some specific examples of the coding process for both types of content analysis. The author has also strengthened the visual content analysis section in terms of how it was coded and how this contributed to research concerns. |
| Reviewer 2 | You might consider adding an **analytical framework** to the Methodology section, offering an overview of your tiered methodology. This framework could clearly present the research focus areas addressed in the Results section and demonstrate how they connect to your methods, analysis, and discussion. | The author has included an analytical framework, Table 1, a tiered methodology and connected it to the results section. |
| Reviewer 1 | **Results:** Clarify key data points: While the breakdown of article numbers is informative, the results would benefit from more precise explanations of why certain newspapers, such as The Daily Mirror, led the coverage. Expanding briefly on potential reasons for the prominence of specific papers would provide deeper insights into the reporting trends. | We have addressed this within the results section but this is hypothesis as opposed to the study itself contributing evidence as to why the Daily Mirror led coverage. |
| Reviewer 1 | **Results:** Link findings to the study's broader implications: After presenting each theme or data point, briefly connect the results to the larger context of media influence on public behaviour during the pandemic. This will help reinforce the study's contribution to agenda-setting theory and understanding panic-buying drivers. | The author has added additional text to each data point and theme briefly connecting back to media influence and agenda setting theory. |
| Reviewer 1 | **Discussion:** Clarify the impact of limitations on the study's conclusions: While the limitations are acknowledged, the discussion could benefit from elaborating on how these limitations might have influenced the results. For instance, explain how excluding social media and other forms of media might affect the generalizability of the findings regarding media influence on panic-buying behaviours. | The author has adjusted this section to include how excluding social media limits the generalisability of the findings and impacts the findings. |
| Reviewer 1 | **Discussion:** Strengthen the linkage between findings and policy recommendations: The section mentions policy implications but could more explicitly connect the study's findings to concrete policy actions. Providing specific examples of how policymakers can utilise the insights from the study to improve communication strategies and food system resilience would enhance the practical relevance of the research. | The discussion section has been rewritten to take the reviewer comments into account. The reviewers will now see that after every pertinent point there is now a linkage to the concrete policy actions and specific examples of how policymakers can use the insight. |
| Reviewer 1 | **Discussion:** Provide a more balanced interpretation of media influence: While the discussion focuses on the media's role in shaping panic-buying behaviour, it could also acknowledge other contributing factors, such as psychological responses to crises or government communication strategies. Incorporating a broader perspective would offer a more nuanced understanding of panic-buying phenomena. | Thank you for this comment, a more balanced conclusive statement has been written towards the end of the discussion section. |
| Reviewer 1 | **Conclusion:** Expand the length and detail of the conclusion: The conclusion is currently too brief and could be expanded to provide a more thorough summary of the essential findings and their implications. It should offer more specific policy recommendations and further emphasise the study's contribution to agenda-setting and framing theory while also acknowledging limitations and areas for future research. This would give the conclusion more weight and reinforce the significance of the study. | Thank you for this useful comment, the authors have rewritten the conclusion and given a summary of findings and implications, and what specific policy recommendations were made. It also outlines the contribution to agenda setting theory, and makes suggestions for further research. |
| Reviewer 2 | **Conclusion:** I would like to read a meaningful concluding section, as well as future research perspectives. | As above. |
